# Novel bionic inspired nanosystem construction for precise delivery of mRNA

**DOI:** 10.3389/fbioe.2023.1160509

**Published:** 2023-03-02

**Authors:** Taihua Yang, Lei Xia, Gen Li, Jie Zhao, Jie Li, Jiahao Ge, Qinggong Yuan, Jianjun Zhang, Kang He, Qiang Xia

**Affiliations:** ^1^ Department of Liver Surgery, Renji Hospital, School of Medicine, Shanghai Jiao Tong University, Shanghai, China; ^2^ Department of Orthopedics, Ruijin Hospital, Shanghai Jiaotong University School of Medicine, Shanghai, China; ^3^ Department of Gastroenterology, Hepatology and Endocrinology, Hannover Medical School, Hannover, Germany; ^4^ Shanghai Engineering Research Center of Transplantation and Immunology, Shanghai, China; ^5^ Shanghai Institute of Transplantation, Shanghai, China

**Keywords:** virus-like mesoporous silica, bionic inspired nanosystem, precise delivery of mRNA, liver target, oral *in Situ* injection

## Abstract

The intracellular delivery of messenger (m)RNA holds great potential for the discovery and development of vaccines and therapeutics. Yet, in many applications, a major obstacle to clinical translation of mRNA therapy is the lack of efficient strategy to precisely deliver RNA sequence to liver tissues and cells. In this study, we synthesized virus-like mesoporous silica (V-SiO_2_) nanoparticles for effectively deliver the therapeutic RNA. Then, the cationic polymer polyethylenimine (PEI) was included for the further silica surface modification (V-SiO_2_-P). Negatively charged mRNA motifs were successfully linked on the surface of V-SiO_2_ through electrostatic interactions with PEI (m@V-SiO_2_-P). Finally, the supported lipid bilayer (LB) was completely wrapped on the bionic inspired surface of the nanoparticles (m@V-SiO_2_-P/LB). Importantly, we found that, compared with traditional liposomes with mRNA loading (m@LNPs), the V-SiO_2_-P/LB bionic-like morphology effectively enhanced mRNA delivery effect to hepatocytes both *in vitro* and *in vivo*, and PEI modification concurrently promoted mRNA binding and intracellular lysosomal escape. Furthermore, m@V-SiO_2_-P increased the blood circulation time (t_1/2_ = 7 h) to be much longer than that of the m@LNPs (4.2 h). Understanding intracellular delivery mediated by the V-SiO_2_-P/LB nanosystem will inspire the next-generation of highly efficient and effective mRNA therapies. In addition, the nanosystem can also be applied to the oral cavity, forehead, face and other orthotopic injections.

## 1 Introduction

mRNA has demonstrated great potential in biomedical applications such as oral and frontal diseases, immunotherapy, regenerative medicine, vaccines, and genetic diseases (Guan and Rosenecker, 2017; Kowalski et al., 2019; Wang and Yu, 2020; Shuai et al., 2021; Xiao et al., 2022). By functioning through translation directly in the cytosol, mRNA achieves higher gene expression efficiency than its counterpart DNA and thus does not carry the risk of DNA-related insertional translation (Granot-Matok et al., 2019). However, exogenous mRNA itself has difficulty penetrating the cytomembranes due to the nature of these negatively charged macromolecules. Moreover, mRNA degrades in seconds when applied alone *in vivo* (Wang HX et al., 2017). mRNA is still subjected to ribonuclease-mediated degradation even though packaged within the nanoparticles (Yen et al., 2018; Dirisala et al., 2019; Dirisala et al., 2022; Lin et al., 2022) Hence, developing an efficient platform to deliver mRNA into target cells for effective translation is very important for the development of mRNA-based therapeutics.

To date, various nanomaterials with different compositions, such as lipid/lipid-like materials, polymers, inorganic materials, and hybrid systems, have been developed for mRNA delivery (Guan and Rosenecker, 2017; Kowalski et al., 2019; Shuai et al., 2021; Xiao et al., 2022). In particular, one of the most developed nanomaterials for mRNA delivery is lipid nanoparticles (LNPs), which have been thoroughly explored. Among these, two have been authorized and successfully applied in the clinic for the delivery of mRNA-based COVID-19 vaccines, mRNA-1273 and BNT162b21 (Anderson et al., 2020; Polack et al., 2020; Baden et al., 2021). Although LNPs are an effective platform for mRNA delivery, the interactions between certain chemical functionalities during storage, such as oxidation, hydrolysis, or transesterification, can lead to the loss of mRNA activity (Fenton et al., 2016; Packer et al., 2021). In addition, physiological barriers confronted by LNPs, such as reaching the target tissues and escaping from endosomes to enter the cytoplasm to further increase the *in vivo* translation efficiency of mRNA molecules, should be overcome (Hou et al., 2021).

Hybrid nanoparticles integrating the advantages of its individual components, such as lipid hybrid nanoparticles, can improve the delivery efficacy of mRNA. For instance, Riley and coauthors developed a library of ionizable polyamine-lipid nanoparticles for *in utero* mRNA delivery to mouse foetuses. These LNPs demonstrate higher efficiency and safety compared to benchmark delivery systems, dilinoleylmethyl-4-dimethylaminobutyrate (DLin-MC3-DMA) and jetPEI, in terms of mRNA delivery to foetal livers, lungs, and intestines (Riley et al., 2021). Virus-like mesoporous silica nanoparticles (MSNs) have shown superior cellular uptake and longer blood circulation times compared with those of conventional MSNs due to their biomimetic morphology (Wang W et al., 2017). Moreover, the surface spikes of virus-like MSNs can provide a continuous open space to bind nucleic acids *via* multivalent interactions and protect them from nuclease degradation, which is beneficial for mRNA delivery (Song et al., 2017; Wang et al., 2018).

**FIGURE 1:**
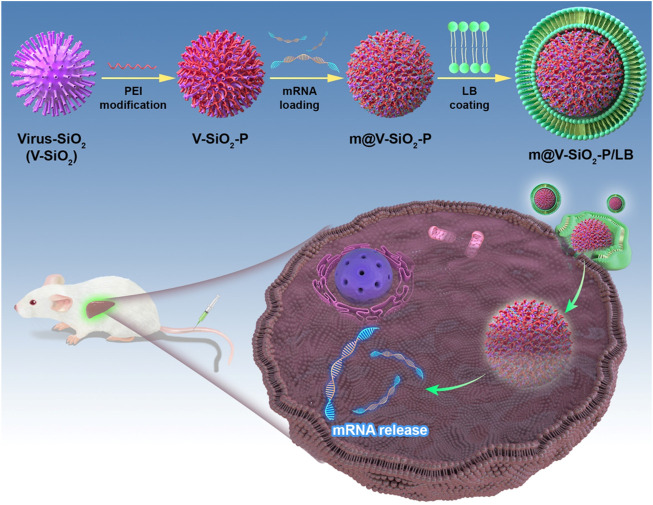
Schematic illustration. Novel bionic inspired nanosystem construction m@V-SiO_2_-P/LB nanosystem for Precise Delivery.

To improve the precise mRNA delivery efficiency, herein, we constructed a mRNA delivery nanoplatform based on virus-like MSNs with surface coating of lipid bilayer (LB). As shown in Figure 1, the described nanosystem (m@V-SiO_2_-P/LB) was successfully constructed by binding HNF4α mRNA onto polyethylenimine (PEI)-modified virus-like MSNs followed by surface coating with a LB. m@V-SiO_2_-P/LB nanoparticles combine the advantages of LNPs, PEI, and virus-like MSNs. First, PEI conjugation contributes to improved mRNA binding and promotes endosome escape (Xia et al., 2009; Wang et al., 2018). Second, their biomimetic morphology enhances gene delivery efficiency both *in vitro* and *in vivo.* Finally, a supported LB coating maintains colloidal stability and effectively prevents mRNA degradation (Noureddine et al., 2020; Gao et al., 2021; Riley et al., 2021; Li et al., 2022). Using green fluorescence protein (GFP)-expressing mRNA as a model, the intracellular delivery and transfection efficiency of m@V-SiO_2_-P/LB nanoparticles were demonstrated to be much higher than that of the benchmark LNPs loaded with mRNA (m@LNPs). Furthermore, *in vivo* fluorescent images demonstrated that more m@V-SiO_2_-P/LB nanoparticles specifically accumulated in the liver, thereby delivering more mRNA to this organ. These findings revealed the improved mRNA delivery performance of the m@V-SiO_2_-P/LB nanoparticles both *in vitro* and *in vivo* compared to LNPs, which could be used in efficient mRNA therapeutics for liver disease treatment.

## 2 Materials and methods

### 2.1 Materials

Hexadecyltrimethylammonium bromide (CTAB), decahydronaphthalene, 1-octadecene (ODE), PEI (branched, *M*_*W*_ 1000), dipalmitoylphosphatidylcholine (DPPC), cholesterol, and 1,2-distearoyl-sn-glycero-3-phosphoethanolamine (DSPE)-PEG were provided by Sigma-Aldrich. NaOH, cyclohexane, NH_4_NO_3_, ammonia aqueous solution (28 wt%), tetraethyl orthosilicate (TEOS), (3-aminopropyl) triethoxysilane (APTES), triethanolamine (TEA), sodium 3-(trihydroxysilyl) propylmethylphosphonate (THPMP), and decahydronaphthalene (98%) were obtained from Macklin Industrial, Inc. LNPs were purchased from Jiliang Pharmaceutical Engineering Co., Ltd. (Shanghai).

### 2.2 Methods

#### 2.1.1 Virus-like MSN (V-SiO_2_) synthesis

V-SiO_2_ was synthesized according to a previous study (Wang W et al., 2017). Briefly, 1.0 g of CTAB was dissolved in 50 ml of water followed by the addition of NaOH (0.1 M, 0.8 ml), and the solution was stirred gently at 60°C. After 2 h, 20 ml of TEOS in cyclohexane (20 v/v %) was added to the mixture with continuous stirring for 48 h. The products were collected by centrifugation, washed with water and ethanol several times, and refluxed in 50 ml of acetone at 50°C overnight to remove the CTAB templates. Then, virus-like MSNs were obtained after centrifugation, washing with ethanol, and drying under vacuum.

#### 2.1.2 PEI modification of V-SiO_2_ (V-SiO_2_-P)

V-SiO_2_-P was prepared according to a previously reported method (Wang et al., 2018). To modify the surface of V-SiO_2_ with PEI, the nanoparticle surface was first modified with phosphonate groups. Typically, 0.1 g of MSNs was dispersed into 30 ml of NaOH solution (pH = 10), and then 40 ml of 60 mM THPMP solution was added before the solution was stirred at 40°C for 2 h. Phosphonate group-modified V-SiO_2_ was obtained by centrifugation and thorough washing, and then the sample was resuspended in 50 ml of carbonate buffer solution (100 mM, pH = 9.6). Next, 150 mg of PEI was added to the solution, which was stirred for 4 h. V-SiO_2_-P was collected by high-speed centrifugation, washed with water, and dried under vacuum.

#### 2.1.3 m@V-SiO_2_-P and m@LNPs preparation and loading efficiency

One microgram of mRNA was mixed with V-SiO_2_-P or LNPs at various weight ratios (V-SiO_2_-P/mRNA = 0, 10, 20, 40, 60, 80) in 0.01 M PBS at 4°C for 30 min. Then, 2 μl of sample was removed for the agarose gel electrophoresis assay (1%, stained with gel safe) at 80 V for 50 min and visualized using a UV transilluminator 2000 (Bio-Rad, Hercules, CA, United States). The rest of each sample was centrifuged at 15,000 rpm for 10 min, from which 2 μl of the supernatant was removed to determine the mRNA content with a Nanodrop 1000 spectrophotometer (Thermo Scientific, Waltham, MA, United States). PBS was used as the blank, and the LNPs were used as controls. Specific information on mRNA and synthesis m@LNPs is further referred to our previous studies (Yang et al., 2022).

#### 2.1.4 LB-coated m@V-SiO_2_-P (m@V-SiO_2_-P/LB) preparation

m@V-SiO_2_-P was prepared at a weight ratio of 10:1 (V-SiO_2_-P:mRNA), and LB coating was added based on a previously published protocol (Meng et al., 2015). A combination of DPPC/cholesterol/DSPE-PEG at a molar ratio of 77.5:20:2.5 was used as the LB layer. The DPPC/cholesterol/DSPE-PEG mixture was suspended in chloroform at a concentration of 10 mg/ml, and the solvent was evaporated using a rotary evaporator to obtain a lipid film. For the LB coating, m@V-SiO_2_-P nanoparticles:LB weight ratio of 1:1.5 (w/w) was selected. Then, the lipid film was added to 0.5 ml of m@V-SiO_2_-P suspension followed by probe sonication for 20 min with a 20/20 s on/off working cycle at a power output of 32.5 W. m@V-SiO_2_-P/LB nanoparticles were collected by centrifugation at 15 000 rpm for 30 min and washed with saline.

### 2.3 Characterization

The morphologies of the m@V-SiO2-P/LB and m@V-SiO2-P nanoparticles were observed *via* transmission electron microscopy (TEM) and scanning electron microscopy (SEM). For TEM observations, samples were suspended in water, dried on a carbon film-supported copper grid, and observed using a JEOL 1010 instrument (JEOL, Tokyo, Japan) operated at 100 kV. For SEM measurements, samples were dropped onto silicon wafers, dried, and characterized using field-emission scanning electron microscopy (FE-SEM) with a JEOL JSM 7800 microscope (JEOL, Tokyo, Japan). The particle size and zeta potential were measured using dynamic light scattering (DLS) with a Zetasizer Nano-ZS instrument.

#### 2.1.5 mRNA release profile from m@LNPs and m@V-SiO_2_-P/LB nanoparticles

The release of mRNA from m@LNPs and m@V-SiO_2_-P/LB nanoparticles was studied by dispersing m@LNPs and m@V-SiO_2_-P/LB nanoparticles (containing 5 μg of mRNA) in 1 ml of 0.01 M PBS (pH = 7.4) at 37°C with shaking at 50 rpm. At predetermined time intervals, samples were centrifuged, and 0.5 ml of supernatant was removed, followed by the addition of the same volume of fresh PBS immediately after sampling. The amount of mRNA released was measured using a Nanodrop.

#### 2.1.6 Cell culture and animals

Human liver L02 cells and human hepatic carcinoma HepG2 cells were provided by ATCC and cultured in high glucose Dulbecco’s modified Eagle’s medium (DMEM) containing 10% foetal bovine serum, streptomycin (100 U ml−1) and penicillin (100 U ml−1) (Invitrogen, CA, United States) in a 37°C incubator with 5% CO_2_ and 95% humidity. Female BALB/c mice (9 weeks old) from Beijing Vital River Laboratory Animal Technology Co. (Beijing, China) were housed in a controlled environment (temperature, 22°C–24°C; humidity, 60%) under a 12-h light-dark cycle. All animal experiments were performed in accordance with the Guide for the Care and Use of Laboratory Animals and approved by the Experimental Animal Ethics Committee in Renji Hospital, School of Medicine, Shanghai Jiao Tong University.

#### 2.1.7 Cellular uptake assay

The cellular uptake efficiency of LNPs and V-SiO_2_-P/LB nanoparticles was studied in L02 (human normal liver) cells. Briefly, L02 cells were seeded in 6-well culture plates at a density of 1 × 10^5^ cells per well and cultured for 24 h. Then, 20 μl of FITC-labelled LNPs or V-SiO_2_-P/LB nanoparticles (100 ng per well) was added to each well for another 3 h of culture. Afterwards, the cells were washed with PBS, stained with DAPI and observed by confocal laser scanning microscopy (Leica HCS A, Leica, Wetzlar, Germany).

#### 2.1.8 mRNA template construction and synthesis

To construct mRNA generation template, GFP sequence was codon optimized and clone into T7 promoter and polyA encoded plasmid. After template linearization, GFP mRNA was generated through *in vitro* transcription kit (Promega) and Cleancap reagent (TriLink). The High performance Liquid Chromatography (HPLC, Agilent 1100, Agilent Technologies, Santa Clara, CA, United States) was applied for mRNA purification. All mRNAs were checked *via* agarose gel electrophoresis and stored frozen at −80°C.

#### 2.1.9 *In vitro* GFP mRNA transfection

The intracellular delivery efficacy of GFP mRNA by the V-SiO_2_-P/LB nanoparticles was evaluated in L02 cells. The L02 cells were seeded in a 12-well plate at the density of 1 × 10^5^ cells per well and cultured for 24 h. One hundred nanograms of free GFP mRNA, GFP mRNA-loaded LNPs or V-SiO_2_-P/LB nanoparticles in PBS was added to each well, and the cells were cultured for 3 h. The cells were then stained with DAPI and subsequently subjected to CLSM observation. For flow cytometry analysis, cells treated with free GFP mRNA, GFP mRNA-loaded LNPs or V-SiO_2_-P/LB nanoparticles were collected after 3 h of incubation, and intracellular green fluorescent protein expression was analysed by flow cytometry (Millipore Guava EasyCyte 5, Millipore, France).

#### 2.1.10 Cytotoxicity and live/dead staining

CCK-8 assays were carried out to evaluate the toxicity of LNPs or V-SiO_2_-P/LB nanoparticles to L02 cells. Briefly, L02 cells were seeded on 96-well plates and incubated with various concentrations of LNPs or V-SiO_2_-P/LB nanoparticles for 24 h. Afterwards, the cell viability was determined *via* a standard CCK-8 protocol.

#### 2.1.11 *In vivo* fluorescence imaging

To investigate the *in vivo* distribution of LNPs or V-SiO_2_-P/LB nanoparticles, mice were intravenously injected with indocyanine green (ICG)-loaded LNPs or V-SiO_2_-P/LB nanoparticles. One, 12, 24, and 48 h after tail vein injection, mice were euthanized and imaged by an *in vivo* imaging system (Xenogen IVIS kinetic, Caliper Life Sciences, United States). Simultaneously, the region of interest (ROI) in each image was analysed by Living Image software 3.2.

#### 2.1.12 *In vivo* mRNA transfection

BALB/c mice were injected with GFP mRNA-loaded LNPs or V-SiO_2_-P/LB nanoparticles *via* the tail vein at a total dose of 1 mg/kg of mRNA per mouse. After 72 h, mice were sacrificed, and liver tissues were cut on ice, stained with DAPI, and evaluated for immunofluorescence.

#### 2.1.13 Acute toxicity test and histopathological examinations

BALB/c mice were randomly divided into three groups (n = 3) and intravenously injected with PBS, LNPs, or V-SiO_2_-P/LB nanoparticles (3 mg/kg, 200 μl) and saline (200 μl) once daily for 7 days. After 7 days of observation, the mice were sacrificed, and the blood and vital organs were collected. Serum was separated from the blood samples, and the levels of cytokines and chemokines, including TNF-α, TNF-γ, IL-1b, IL-2, IL-3, IL-4, IL-10, IL-12p70, IL-13, and IL-17, were determined by a multiplex protein array kit according to the manufacturer’s protocols (Bio-Rad Laboratories, CA). All cytokines and chemokine levels were corrected according to our previously reported method (Yang et al., 2021). Vital organs were fixed, embedded in paraffin, sectioned, and stained with haematoxylin and eosin (H&E). The H&E-stained sections were observed under a light microscope (LeicaDMi8M, Leica, Wetzlar, Germany) for histopathological examination.

#### 2.1.14 Pharmacokinetic analysis

BALB/c mice were injected with ICG-labelled LNPs or V-SiO_2_-P/LB (4 mg/kg, i. v.). Blood was collected from the ocular vein at 5 min, 1, 2, 4, 8, 12, and 24 h after injection, and the fluorescence intensity at 780 nm was detected by a multifunctional microplate reader (SpectraMax M5, Molecular Devices, United States).

#### 2.1.15 Statistical analysis

Statistical analyses were carried out using two-sided Student’s t test for unpaired comparisons with GraphPad Prism. Statistical significance is depicted with error bars representing ±SD. A *p*-value <0.05 was considered significant.

## 3 Results and discussion

### 3.1 The optimal design and characterization of the m@V-SiO_2_-P/LB nanoparticles

**FIGURE 2:**
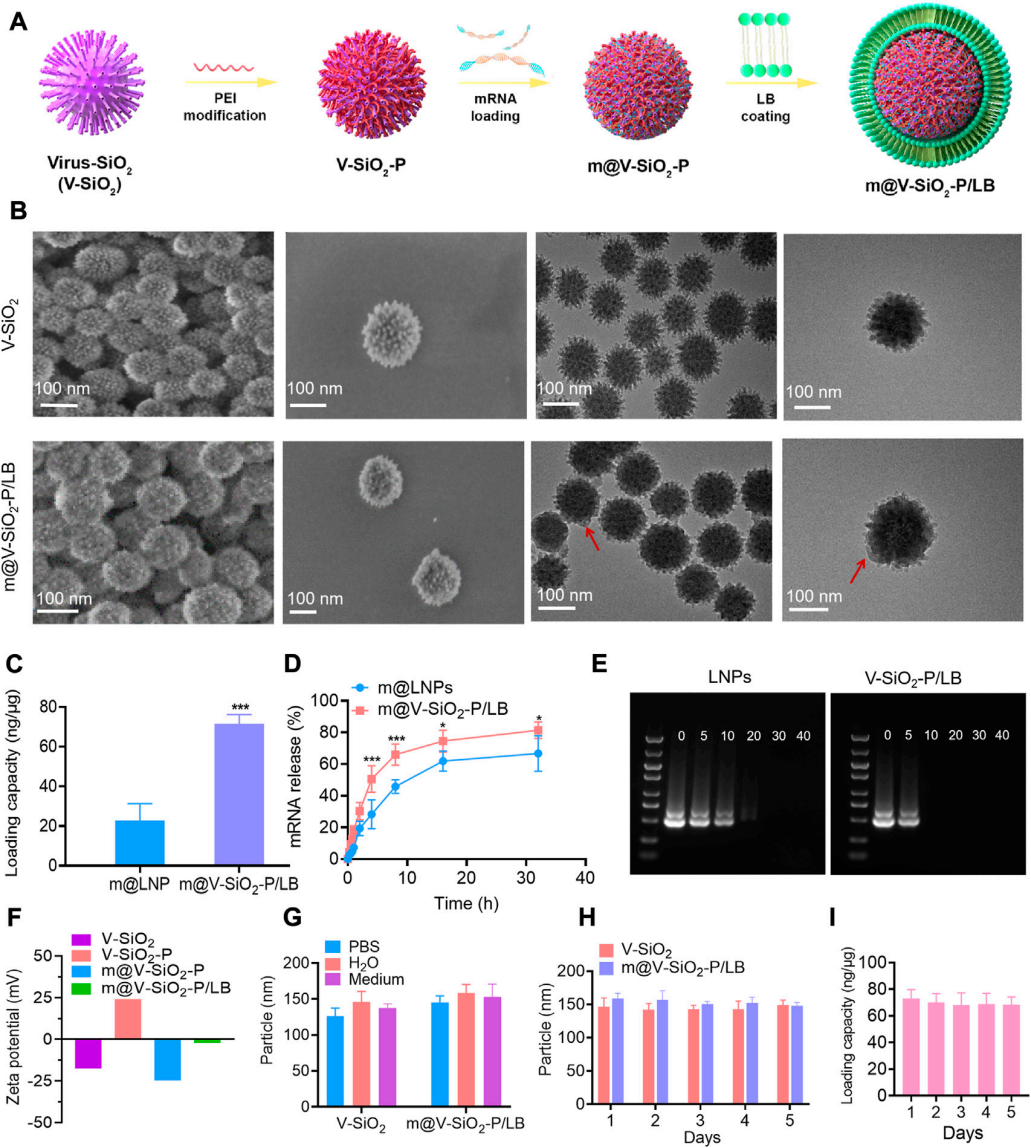
The optimal design and characterization of m@V-SiO_2_-P/LB nanoparticles. **(A)** An illustration of the V-SiO_2_-P/LB nanotopography for precise mRNA delivery. **(B)** Structural characterization of V-SiO_2_ and m@V-SiO_2_-P/LB by SEM and TEM. **(C)** GFP mRNA loading capacities of LNPs and V-SiO_2_-P/LB nanoparticles. **(D)** Cumulative release of GFP mRNA from LNPs and V-SiO_2_-P/LB nanoparticles at pH 7.4. *p < 0.05, **p < 0.001. **(E)** Agarose gel electrophoresis of the GFP mRNA-LNPs and GFP m@V-SiO_2_-P/LB complexes, respectively. The amounts of LNPs and V-SiO_2_-P/LB varied from 0 to 40 μg, while the amount of mRNA was kept at a constant (0.5 μg). **(F)** Zeta potentials of V-SiO_2_ and PEI-V-SiO_2_ (V-SiO_2_-P), GFP mRNA loading, and LB coating. **(G)** Size distribution analysis of V-SiO_2_ and m@V-SiO_2_-P/LB nanoparticles dispersed in water, PBS and cell culture medium for 24 h. **(H)** Particle size distribution curves of V-SiO_2_ and V-SiO_2_-P/LB nanoparticles after suspension in PBS for 1, 2, 3, 4, and 5 days measured by DLS. **(I)** mRNA loading by m@V-SiO_2_-P/LB nanoparticles after suspension in PBS for 1, 2, 3, 4 and 5 days.

According to our design, the stepwise procedure for the fabrication of the m@V-SiO_2_-P/LB nanoparticles is illustrated in Figure 2A. Virus-like mesoporous silica nanoparticles (V-SiO_2_) were synthesized in a two-phase reaction system (water/cyclohexane) with a low template concentration (CTAB) (Wang W et al., 2017). The scanning electron microscopy (SEM) and transmission electron microscopy (TEM) images of the V-SiO_2_ show that they had a one-of-a kind bionic inspired morphology, all nanoparticles were well dispersed, and presented with a uniform size distribution that can be determined as approximately −115 nm (Figure 2B, top). Simultaneously, hydration radius was further confirmed by dynamic light scattering (DLS), which was slightly increased to 120 nm (Supplementary Figure S1A). The prepared SiO_2_ nanoparticles were immediately modified with PEI (V-SiO_2_-P), mRNA loading followed by the addition of a complete LB coating (V-SiO_2_-P/LB; The thickness of LB is 7–10 nm; Red arrow). The two steps of PEI and LB modifications could improve the mRNA binding efficiency, facilitate lysosomal escape, and maintain colloidal stability that is capable of effectively avoiding mRNA degradation (Wang et al., 2018) (Noureddine et al., 2020) (Xia et al., 2009; Wang et al., 2018) (Noureddine et al., 2020; Gao et al., 2021; Riley et al., 2021; Li et al., 2022). At present, there are few studies on mRNA delivery by inorganic nanomaterials, and LNPs have been an effective mRNA delivery (Meng et al., 2015). Therefore, subsequently, we chose commercial LNPs that have been previously reported as a control (Akinc and Battaglia, 2013; Turnbull et al., 2016) to compare mRNA loading and release. The mRNA loading into the LNPs was calculated as 22.74 ng/μg, remarkably lower than that of the m@V-SiO_2_-P/LB which was determined as 71.52 ng/μg (Figure 2C). The low mRNA loading efficiency of LNPs may be due to the low concentration of phospholipid, but high concentration of phospholipid will lead to poor morphology and stability of LNPs (Robinson et al., 2018). We then performed further mRNA release efficiency of the two nanocarriers at pH 7.4. In Figure 2D, at 8 h, the mRNA release rate in the m@V-SiO_2_-P/LB group was 65.94%, significantly higher than that of m@LNPs group (45.77%). The mRNA release rate in the m@V-SiO_2_-P/LB group was 81.4% at 32 h, while it was 66.72% in the m@LNPs group at the same time point. All these results suggest that the V-SiO_2_-P/LB nanoparticles increased mRNA loading efficiency due to their bionic inspired nanospikes and PEI modification, but the release trend of mRNA was identical with LNPs. To further investigate the binding affinity of mRNA to the LNPs or V-SiO_2_-P/LB nanoparticles, gel retardation experiments were then performed (Figure 2E). The doses of LNPs and V-SiO_2_-P/LB were ranged from 0 to 40 μg, and the concentration of mRNA was kept constant (0.5 μg). In the LNPs group, the intensity of the offset mRNA bands decreased with increasing LNPs concentration. When the LNPs concentration was 5 μg, the LNPs bands were still brighter than that of V-SiO_2_-P/LB, further indicating that the mRNA was completely bound to the V-SiO_2_-P/LB nanoparticles.

Then m@V-SiO_2_-P/LB were observed under both SEM and TEM. As displayed in Figure 2B (bottom), uniform coating of the surfaces with an intact LB can be successfully found. Furthermore, the changes in zeta potential were used to characterize the success of PEI modification, mRNA loading and LB coating. As shown in Figure 2F, the original charge of V-SiO_2_ was determined as −17.4 mV, and after PEI modification, V-SiO_2_-P exhibited a surface charge of +24 mV, indicating that PEI was successfully bound to the bionic inspired surface. After anchoring the negatively charged exogenous GFP mRNA, the zeta potential of V-SiO_2_-P reversed to approximately −12 mV, indicating that the mRNA was mostly covered on the rough surface of the Si based nanoparticles. Finally, the zeta potential became electrically neutral (−2 mV) after LB coating, primarily demonstrating the successful wrapping of lipid layer. Moreover, the particle sizes of V-SiO_2_, V-SiO_2_-P, m@V-SiO_2_-P and m@V-SiO_2_-P/LB and their stability in different media were further analysed by DLS. As shown in Supplementary Figure S1, the particle size after PEI modification, mRNA loading and LB coating increased from 120 to 122 nm, 124, and 132 nm, respectively. The particle size distributions are similar after dispersed V-SiO_2_ or m@V-SiO_2_-P/LB in water, PBS and cell culture medium (Figure 2G). In addition, V-SiO_2_ and m@V-SiO_2_-P/LB suspensions were stored in PBS for 1, 2, 3, 4, and 5 d. As shown in Figure 2H, and all size distributions have no significant changes even after 5 d incubation. Meanwhile, no obvious aggregation or sedimentation was observed, indicating that the fabricated m@V-SiO_2_-P/LB had exceptional stability. As expected, the amount of loaded mRNA did not change after 5 days (Figure 2H). Meng et al. also confirmed by repeated experiments that there was no leakage, and the nanoparticles remained intact for at least 1 month after LB coating (Meng et al., 2015).

### 3.2*In vitro* cytotoxicity assay, cellular internalization, and GFP mRNA transfection

**FIGURE 3:**
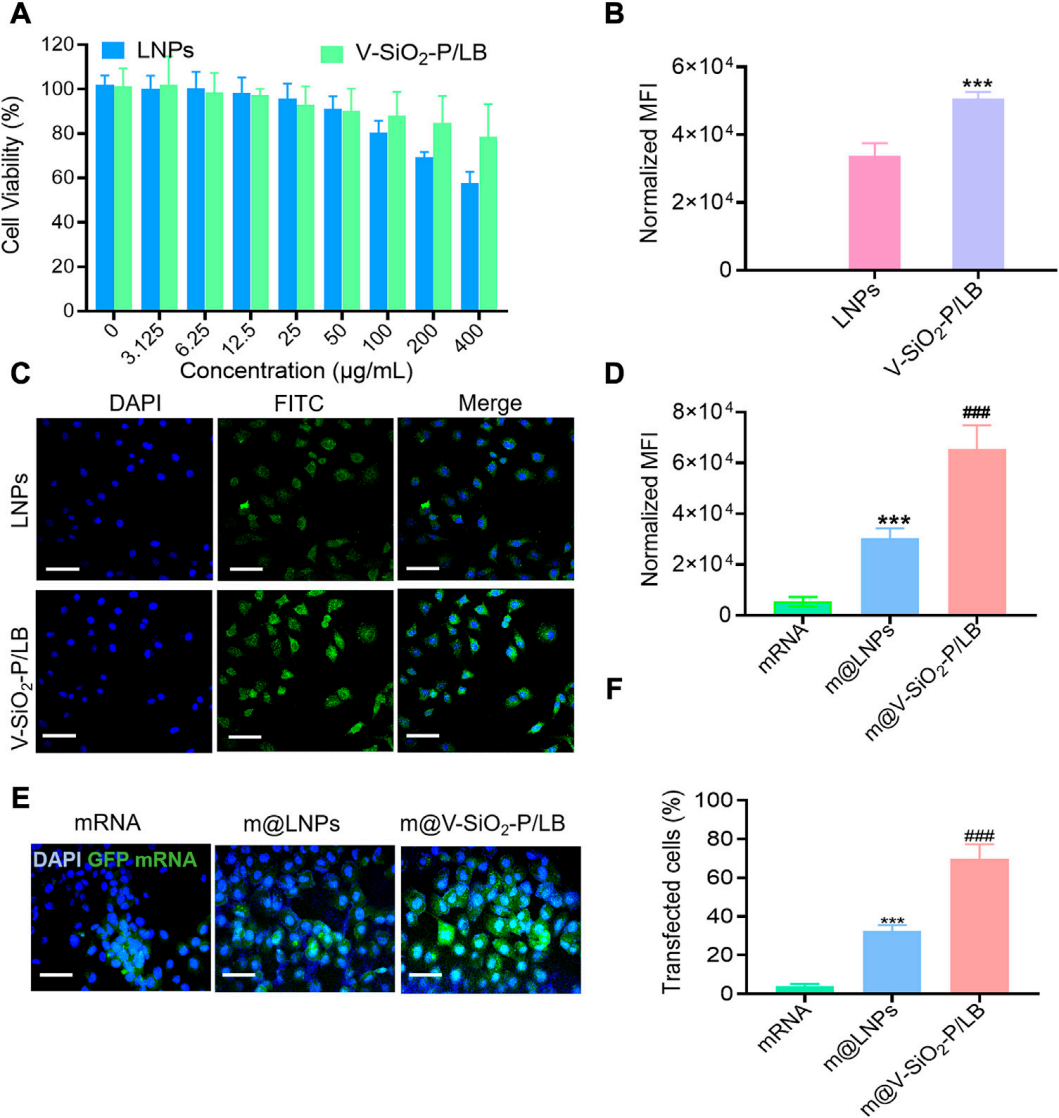
***In vitro* cellular internalization and GFP mRNA transfection. (A)** Viability of L02 cells treated with LNPs or V-SiO_2_-P/LB nanoparticles at concentrations ranging from 0 to 800 μg/mL for 24 h. **(B)** Quantification of FITC fluorescence in L02 cells treated with FITC-labelled NP or V-SiO_2_-P/LB nanoparticles (100 ng per well, 3 h, *n* = 3). ***p < 0.001. **(C)** Confocal laser scanning microscopy (CLSM) observations of L02 cells after incubation with LNPs and V-SiO_2_-P/LB nanoparticles for 6 h. Scale bar: 50 μm. **(D)** Quantification of green fluorescence intensity (indicating GFP expression level) in L02 cells treated with free GFP mRNA, m@LNPs, m@V-SiO_2_-P/LB nanoparticles (100 ng per well, 3 h, *n* = 3). ***p < 0.001 vs. mRNA, ###*p* < 0.001 vs. m@LNPs. **(E)** CLSM confocal microscopy images of GFP expression in L02 cells treated with free GFP mRNA, m@LNPs, m@V-SiO_2_-P/LB. **(F)** The effect of LNPs and V-SiO_2_-P/LB nanoparticles on the transfection of GFP detected by flow cytometry.

An ideal intracellular mRNA delivery nanocarriers must present outstanding biocompatibility. Advancedly, the cytotoxicity of the LNPs and V-SiO_2_-P/LB was determined by CCK-8 assay after co-incubated with L02 cells (normal human liver cells) for 6h, respectively. Figure 3A shows that both the LNPs and V-SiO_2_-P/LB nanoparticles exhibit dose-dependent cytotoxic behaviour toward L02 cells. The cell mortality rate was less than 20% when the concentration of V-SiO_2_-P/LB nanoparticles ranged from 5 to 400 μg/ml. Exhilaratingly, when the concentration of LNPs reached 400 μg/ml, cell viability was only calculated as 57%, further verifying the advantageous of our bionic inspired nanoparticles. In addition to exceptional biocompatibility, an optimal mRNA delivery nanovector needs to precisely deliver mRNA motifs into living cells and achieve high transfection efficiency. Therefore, endocytosis effect of L02 cells toward FITC-labelled LNPs and V-SiO_2_-P/LB nanoparticles along with nuclear staining with DAPI was carefully studied. The confocal laser scanning microscopy (CLSM) results displayed that the relative cellular uptake rates of the LNPs and V-SiO_2_-P/LB nanoparticles were very different (Figure 3C). After 3 h of incubation, both the LNPs and V-SiO_2_-P/LB nanoparticles were able to penetrate the cell membrane and accumulate in the cytoplasm. Satisfactorily, the cellular uptake rate of the V-SiO_2_-P/LB nanoparticles was obviously higher than that of the LNPs. The same trend was also observed by quantitative analysis of the median fluorescence intensity (MFI) by flow cytometry (Figure 3B). This is mainly due to the rough virus-like morphology promotes cellular uptake and has a unique internalization pathway (Niu et al., 2013; Wang W et al., 2017). Finally, the expression level of GFP was also observed by CLSM with fluorescein-labelled mRNA. L02 cells were incubated with m@LNPs or m@V-SiO_2_-P/LB nanoparticles for 24 h, and free mRNA was used as a control. The results are shown in Figure 3E, where the intensity of green fluorescence, indicating the GFP expression level, was weak in the free mRNA group, while the intensity of green fluorescence was stronger in m@V-SiO_2_-P/LB nanoparticle group. The quantified flow cytometry results were consistent with the CLSM data (Figure 3D), with the m@V-SiO_2_-P/LB nanoparticles showing the highest MFI; this was due to its strong binding affinity and high cellular uptake, benefiting from the PEI modification and rough virus-like morphology, respectively. In the meantime, the transfection efficiency in the m@V-SiO_2_-P/LB nanoparticle group was 69.97%, which was relatively higher than that in the LNPs group (32.61%). In brief, our LB coated bionic inspired nanocarriers have superior capabilities of precise mRNA delivery toward liver cells and excellent mRNA transfection effect.

### 3.3V-SiO_2_-P/LB nanoparticles contribute to effective mRNA delivery to the liver *in vivo*

**FIGURE 4:**
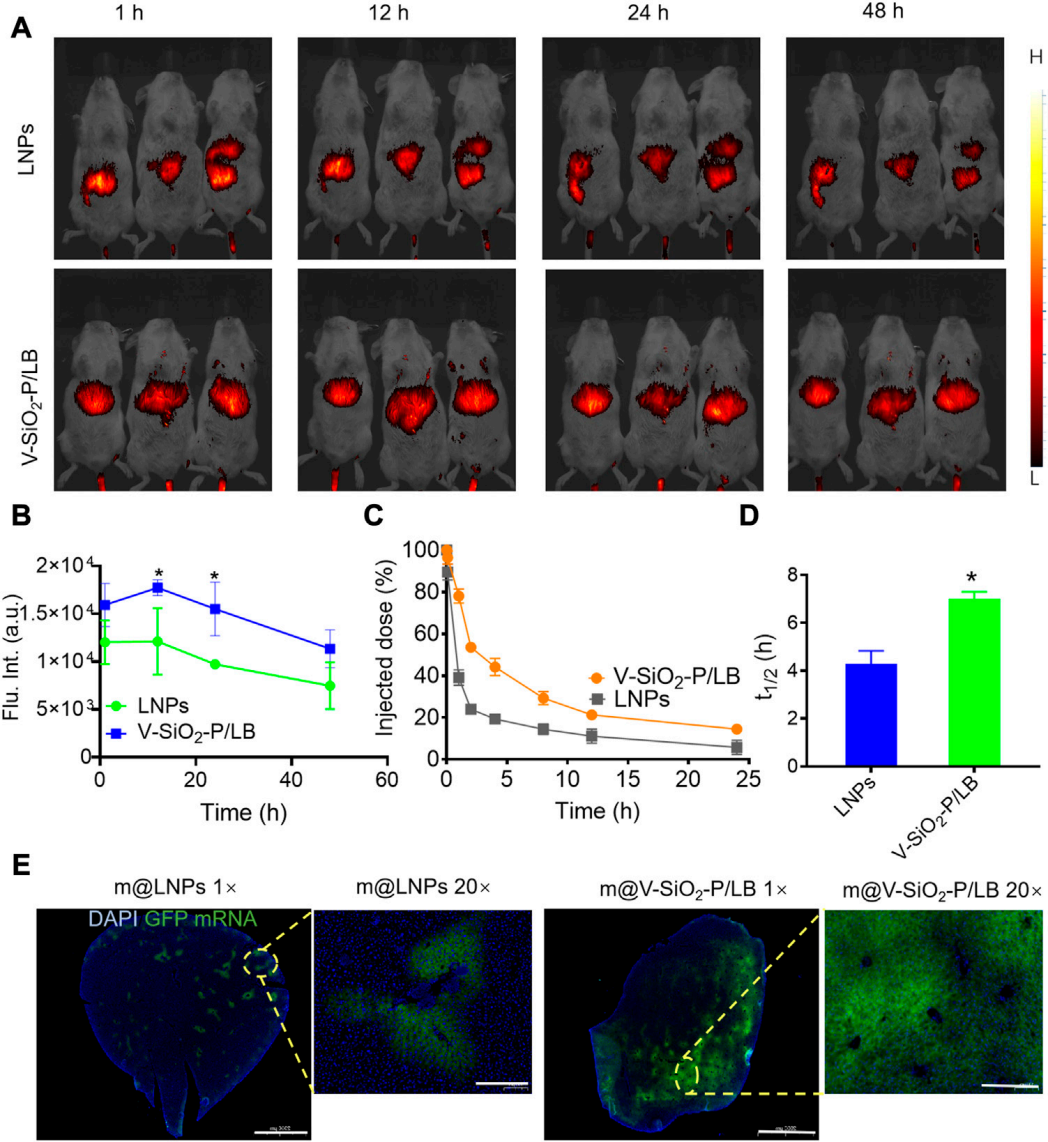
**V-SiO**_**2**_ **-P/LB nanoparticles contribute to precise mRNA delivery to the liver *in vivo*. (A)** Bioluminescence analyses at 1 h, 12 h, 24 h, 48 h and 72 h in BALB/c mice (n = 3 mice per group) injected **(I)**. v. with ICG-labelled LNPs or V-SiO_2_-P/LB nanoparticles. **(B)** Quantification of light intensity over time. **(C)** Time-dependent blood levels of ICG-labelled LNPs and V-SiO_2_-P/LB nanoparticles after tail vein injection, calculated as the percentage of injected dose remaining in the blood. **(D)** Blood circulation half-lives (t_1/2_) of LNPs and V-SiO_2_-P/LB nanoparticles (n = 3 mice per group). *p < 0.05. **(E)** GFP mRNA expression levels in liver tissue after m@LNPs or m@v-SiO2-P/LB treatment under different magnification.

After demonstrating the high efficiency of mRNA delivery by the V-SiO_2_-P/LB nanoparticles to liver cells *in vitro*, we were encouraged to investigate the efficacy of the V-SiO_2_-P/LB nanoparticles to deliver mRNA to the liver and their biosafety *in vivo*. First, the biodistribution (liver-targeting ability) of LNPs and V-SiO_2_-P/LB nanoparticles was performed using fluorescence imaging system. Indocyanine green (ICG)-labelled LNPs and V-SiO_2_-P/LB nanoparticles were injected into BALB/c mice through the tail vein, and fluorescence images were collected at different time points (1, 12, 24, and 48 h). As expected, fluorescence signals were detected in the livers in both the LNP- and V-SiO_2_-P/LB nanoparticle-treated groups 1 h after injection (Figures 4A, B; Supplementary Figure S2). It is worth noting that the fluorescence in the LNP and V-SiO_2_-P/LB groups peaked at 12 h after injection. Apparently, the liver fluorescence signal in the V-SiO_2_-P/LB group was prominently higher than that in the LNP group at all time points (Figure 4B). Additionally, long blood circulation is particularly important for mRNA delivery *in vivo*. Compared with the blood circulation half-life (t_1/2_) of the LNPs with only 4.2 h, that of the V-SiO_2_-P/LB nanoparticles was lengthened to 7 h (Figures 4C, D). The prolonged blood circulation of the V-SiO_2_-P/LB nanoparticles may promote the partial mRNA transport period and aggregation duration in the liver, probably due to its special surface morphology and outstanding stability in serum, which is consistent with previous reports (Hu et al., 2013).

Immediately, m@LNPs and m@V-SiO_2_-P/LB nanoparticles were injected into healthy BALB/c mice *via* the tail vein, and the livers were removed 12 h later for sectioning (in the dark on ice) and DAPI staining. A tissue immunofluorescence scan was used to observe the expression level of GFP. As shown in Figure 4E, the livers tissue in the m@LNP group showed faint green fluorescence, while the livers tissue of the mice after the m@V-SiO_2_-P/LB treatment presented large areas of green fluorescence with prominently higher signal intensity. These results also confirmed that the V-SiO_2_-P/LB nanoparticles can efficiently delivered mRNA to the liver tissue.

### 3.4 Biosafety of V-SiO_2_-P/LB nanoparticles

**FIGURE 5:**
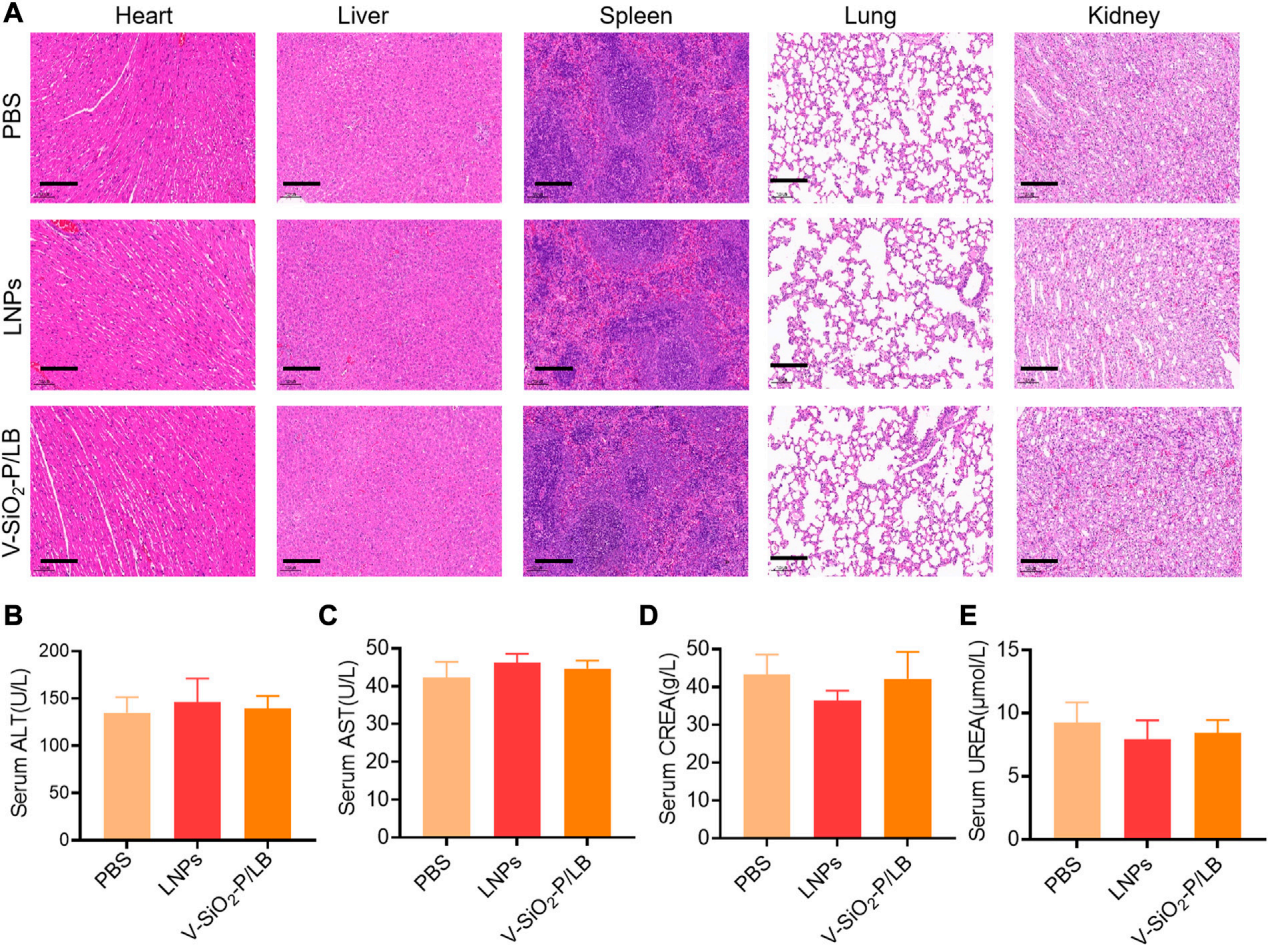
**Biosafety of V-SiO**_**2**_ **-P/LB nanoparticles *in vivo*. (A)** Histopathological examination of vital organ tissues after intravenous injection of PBS, LNPs, and V-SiO_2_-P/LB nanoparticles (3 mg/kg, 200 μL) and saline (200 μL) once daily for 7 days by H&E staining. Scale bars, 200 μm. n = 3 mice per group. Detection of serum **(B)** ALT, **(C)** AST, **(D)** CREA and **(E)** UREA levels after the acute toxicity test. n = 3 mice per group.

**FIGURE 6:**
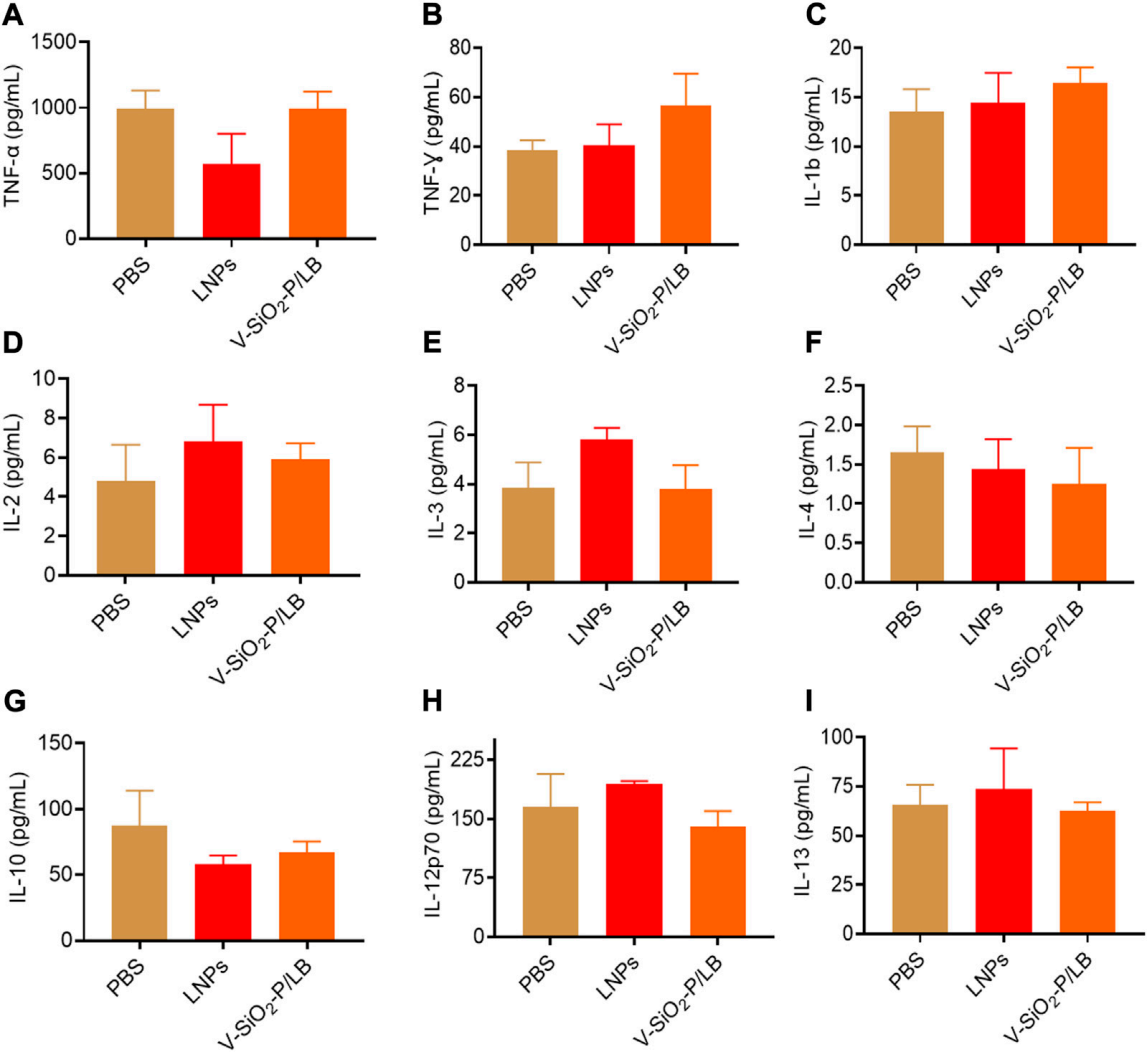
**The cytokines and chemokines of V-SiO**_**2**_ **-P/LB nanoparticles *in vivo*.** The levels of the cytokines and chemokines **(A)** TNF-α, **(B)** TNF-γ, **(C)** IL-1b, **(D)** IL-2, **(E)** IL-3, **(F)** IL-4, **(G)** IL-10, **(H)** IL-12p70, and **(I)** IL-13 determined by a multiplex protein array kit.

Before the potential clinical translation, we examined the acute toxicity of a high dose (3 mg/kg) of V-SiO_2_-P/LB nanoparticles in mice after intravenous administration once daily for 7 days. Fortunately, no death or severe weight loss were observed in both LNPs and V-SiO_2_-P/LB treated groups. Histopathological examination showed no obvious damage or abnormalities in the liver and kidney in all three groups (Figure 5A). In addition, the serum biochemical indices (AST, ALT, CREA and UREA) in the LNP and V-SiO_2_-P/LB groups did not change significantly compared with the PBS blank group, indicating that both the LNPs and V-SiO_2_-P/LB nanoparticles are highly biocompatible (Figures 5B–D). Notably, we did not find any fluctuations in the levels of cytokines or chemokines in the blood between the groups (Figure 6), indicating that the V-SiO_2_-P/LB nanoparticles had a satisfying biosafety profile. To date, we have only demonstrated the ability of this delivery system to load GFP mRNA and target the liver in normal hepatocytes and in mice. Therefore, we further explore the functional mRNA loading ability, mRNA integrity, targeting ability and therapeutic effect of this antiviral nano-delivery system by constructing different liver disease models.

## 4 Conclusion

In this work, we have successfully developed V-SiO_2_-P/LB nanocarrier for the first time and it can efficiently deliver mRNA to the liver tissue. In this novel nanoplatform, PEI was uniformly modified on the surface of virus-like mesoporous silica nanoparticles, and LB was completely coated on this surface. V-SiO_2_-P/LB nanoparticles had excellent biocompatibility compared with LNPs, especially the more excellent mRNA delivering capability both *in vitro* and *in vivo*. The rough bionic inspired surface and PEI modification of the silica nanoparticles can sustainably increase loading and binding capacity of GFP mRNA (71.52 ng/μg), and the unique physical morphology enhanced hepatocyte endocytosis efficiency. Meanwhile, LB effectively prevented mRNA degradation and enhanced the blood circulation period of mRNA. Thus, the present study demonstrates that V-SiO_2_-P/LB nanoparticles with satisfied biosafety have great potential to effectively deliver mRNA.

## Data Availability

The raw data supporting the conclusions of this article will be made available by the authors, without undue reservation.

## References

[B1] Akinc A. Battaglia G. (2013). Exploiting endocytosis for nanomedicines. Cold Spring Harb. Perspect. Biol. 5 (11), a016980. 10.1101/cshperspect.a016980 24186069 PMC3809578

[B2] Anderson E. J. Rouphael N. G. Widge A. T. Jackson L. A. Roberts P. C. Makhene M. (2020). Safety and immunogenicity of SARS-CoV-2 mRNA-1273 vaccine in older adults. N. Engl. J. Med. 383 (25), 2427–2438. 10.1056/NEJMoa2028436 32991794 PMC7556339

[B3] Baden L. R. El Sahly H. M. Essink B. Kotloff K. Frey S. Novak R. (2021). Efficacy and safety of the mRNA-1273 SARS-CoV-2 vaccine. N. Engl. J. Med. 384 (5), 403–416. 10.1056/NEJMoa2035389 33378609 PMC7787219

[B4] Dirisala A. Uchida S. Li J. Van Guyse J. F. R. Hayashi K. Vummaleti S. V. C. (2022). Effective mRNA protection by poly(l-ornithine) synergizes with endosomal escape functionality of a charge-conversion polymer toward maximizing mRNA introduction efficiency. Macromol. Rapid Commun. 43 (12), e2100754. 10.1002/marc.202100754 35286740

[B5] Dirisala A. Uchida S. Tockary T. A. Yoshinaga N. Li J. Osawa S. (2019). Precise tuning of disulphide crosslinking in mRNA polyplex micelles for optimising extracellular and intracellular nuclease tolerability. J. Drug Target 27 (5-6), 670–680. 10.1080/1061186X.2018.1550646 30499743

[B6] Fenton O. S. Kauffman K. J. McClellan R. L. Appel E. A. Dorkin J. R. Tibbitt M. W. (2016). Bioinspired alkenyl amino alcohol ionizable lipid materials for highly potent *in vivo* mRNA delivery. Adv. Mater 28 (15), 2939–2943. 10.1002/adma.201505822 26889757 PMC5245883

[B7] Gao Y. Men K. Pan C. Li J. Wu J. Chen X. (2021). Functionalized DMP-039 hybrid nanoparticle as a novel mRNA vector for efficient cancer suicide gene therapy. Int. J. Nanomedicine 16, 5211–5232. 10.2147/IJN.S319092 34366664 PMC8335320

[B8] Granot-Matok Y. Kon E. Dammes N. Mechtinger G. Peer D. (2019). Therapeutic mRNA delivery to leukocytes. J. Control Release 305, 165–175. 10.1016/j.jconrel.2019.05.032 31121277

[B9] Guan S. Rosenecker J. (2017). Nanotechnologies in delivery of mRNA therapeutics using nonviral vector-based delivery systems. Gene Ther. 24 (3), 133–143. 10.1038/gt.2017.5 28094775

[B10] Hou X. Zaks T. Langer R. Dong Y. (2021). Lipid nanoparticles for mRNA delivery. Nat. Rev. Mater 6 (12), 1078–1094. 10.1038/s41578-021-00358-0 34394960 PMC8353930

[B11] Hu X. Hu J. Tian J. Ge Z. Zhang G. Luo K. (2013). Polyprodrug amphiphiles: Hierarchical assemblies for shape-regulated cellular internalization, trafficking, and drug delivery. J. Am. Chem. Soc. 135 (46), 17617–17629. 10.1021/ja409686x 24160840

[B12] Kowalski P. S. Rudra A. Miao L. Anderson D. G. (2019). Delivering the messenger: Advances in technologies for therapeutic mRNA delivery. Mol. Ther. 27 (4), 710–728. 10.1016/j.ymthe.2019.02.012 30846391 PMC6453548

[B13] Li Z. Zhang X. Q. Ho W. Bai X. Jaijyan D. K. Li F. (2022). Lipid-polymer hybrid "Particle-in-Particle" nanostructure gene delivery platform explored for lyophilizable DNA and mRNA COVID-19 vaccines. Adv. Funct. Mater 32 (40), 2204462. 10.1002/adfm.202204462 35942271 PMC9349454

[B14] Lin Y. Wagner E. Lachelt U. (2022). Non-viral delivery of the CRISPR/cas system: DNA versus RNA versus RNP. Biomater. Sci. 10 (5), 1166–1192. 10.1039/d1bm01658j 35103261

[B15] Meng H. Wang M. Liu H. Liu X. Situ A. Wu B. (2015). Use of a lipid-coated mesoporous silica nanoparticle platform for synergistic gemcitabine and paclitaxel delivery to human pancreatic cancer in mice. ACS Nano 9 (4), 3540–3557. 10.1021/acsnano.5b00510 25776964 PMC4415452

[B16] Niu Y. Yu M. Hartono S. B. Yang J. Xu H. Zhang H. (2013). Nanoparticles mimicking viral surface topography for enhanced cellular delivery. Adv. Mater. Deerf. Beach, Fla) 25 (43), 6233–6237. 10.1002/adma.201302737 23946251

[B17] Noureddine A. Maestas-Olguin A. Saada E. A. LaBauve A. E. Agola J. O. Baty K. E. (2020). Engineering of monosized lipid-coated mesoporous silica nanoparticles for CRISPR delivery. Acta Biomater. 114, 358–368. 10.1016/j.actbio.2020.07.027 32702530

[B18] Packer M. Gyawali D. Yerabolu R. Schariter J. White P. (2021). A novel mechanism for the loss of mRNA activity in lipid nanoparticle delivery systems. Nat. Commun. 12 (1), 6777. 10.1038/s41467-021-26926-0 34811367 PMC8608879

[B19] Polack F. P. Thomas S. J. Kitchin N. Absalon J. Gurtman A. Lockhart S. (2020). Safety and efficacy of the BNT162b2 mRNA covid-19 vaccine. N. Engl. J. Med. 383 (27), 2603–2615. 10.1056/NEJMoa2034577 33301246 PMC7745181

[B20] Riley R. S. Kashyap M. V. Billingsley M. M. White B. Alameh M. G. Bose S. K. (2021). Ionizable lipid nanoparticles for *in utero* mRNA delivery. Sci. Adv. 7 (3), eaba1028. 10.1126/sciadv.aba1028 33523869 PMC7806221

[B21] Robinson E. MacDonald K. D. Slaughter K. McKinney M. Patel S. Sun C. (2018). Lipid nanoparticle-delivered chemically modified mRNA restores chloride secretion in cystic fibrosis. Mol. Ther. 26 (8), 2034–2046. 10.1016/j.ymthe.2018.05.014 29910178 PMC6094356

[B22] Shuai Q. Zhu F. Zhao M. Yan Y. (2021). mRNA delivery via non-viral carriers for biomedical applications. Int. J. Pharm. 607, 121020. 10.1016/j.ijpharm.2021.121020 34416327

[B23] Song H. Yu M. Lu Y. Gu Z. Yang Y. Zhang M. (2017). Plasmid DNA delivery: Nanotopography matters. J. Am. Chem. Soc. 139 (50), 18247–18254. 10.1021/jacs.7b08974 29151352

[B24] Turnbull I. C. Eltoukhy A. A. Fish K. M. Nonnenmacher M. Ishikawa K. Chen J. (2016). Myocardial delivery of lipidoid nanoparticle carrying modRNA induces rapid and transient expression. Mol. Ther. J. Am. Soc. Gene Ther. 24 (1), 66–75. 10.1038/mt.2015.193 PMC4754552 26471463

[B25] Wang Y. Song H. Yu M. Xu C. Liu Y. Tang J. (2018). Room temperature synthesis of dendritic mesoporous silica nanoparticles with small sizes and enhanced mRNA delivery performance. J. Mater Chem. B 6 (24), 4089–4095. 10.1039/c8tb00544c 32255152

[B26] Wang H. X. Li M. Lee C. M. Chakraborty S. Kim H. W. Bao G. (2017). CRISPR/Cas9-Based genome editing for disease modeling and therapy: Challenges and opportunities for nonviral delivery. Chem. Rev. 117 (15), 9874–9906. 10.1021/acs.chemrev.6b00799 28640612

[B27] Wang W. Wang P. Tang X. Elzatahry A. A. Wang S. Al-Dahyan D. (2017). Facile synthesis of uniform virus-like mesoporous silica nanoparticles for enhanced cellular internalization. ACS central Sci. 3 (8), 839–846. 10.1021/acscentsci.7b00257 PMC5571464 28852697

[B28] Wang Y. Yu C. (2020). Emerging concepts of nanobiotechnology in mRNA delivery. Angew. Chem. Int. Ed. Engl. 59 (52), 23374–23385. 10.1002/anie.202003545 32400110

[B29] Xia T. Kovochich M. Liong M. Meng H. Kabehie S. George S. (2009). Polyethyleneimine coating enhances the cellular uptake of mesoporous silica nanoparticles and allows safe delivery of siRNA and DNA constructs. ACS Nano 3 (10), 3273–3286. 10.1021/nn900918w 19739605 PMC3900639

[B30] Xiao Y. Tang Z. Huang X. Chen W. Zhou J. Liu H. (2022). Emerging mRNA technologies: Delivery strategies and biomedical applications. Chem. Soc. Rev. 51 (10), 3828–3845. 10.1039/d1cs00617g 35437544

[B31] Yang T. Poenisch M. Khanal R. Hu Q. Dai Z. Li R. (2022). Corrigendum to 'Therapeutic HNF4A mRNA attenuates liver fibrosis in a preclinical model' [J Hepatol (2021) 1420-1433]. J. Hepatol. 77 (1), 270. 10.1016/j.jhep.2022.03.023 35397937

[B32] Yang T. Poenisch M. Khanal R. Hu Q. Dai Z. Li R. (2021). Therapeutic HNF4A mRNA attenuates liver fibrosis in a preclinical model. J. Hepatol. 75 (6), 1420–1433. 10.1016/j.jhep.2021.08.011 34453962

[B33] Yen A. Cheng Y. Sylvestre M. Gustafson H. H. Puri S. Pun S. H. (2018). Serum nuclease susceptibility of mRNA cargo in condensed polyplexes. Mol. Pharm. 15 (6), 2268–2276. 10.1021/acs.molpharmaceut.8b00134 29672061

