# Supplementary material for: Novel bionic inspired nanosystem construction for precise delivery of mRNA
Source: Front Bioeng Biotechnol. 2023 Mar 2;11:1160509. doi: 10.3389/fbioe.2023.1160509 (PMC10018395; doi:10.3389/fbioe.2023.1160509)
Supplement: Supplementary file 1 [file DataSheet1.docx]

Supplementary Material

Novel Bionic Inspired Nanosystem Construction for Precise Delivery of mRNA

Taihua Yang^1*^, Lei Xia^1*^, Gen Li^2*^, Jie Zhao^1^, Jie Li^1^, Jiahao Ge^1^, Qinggong Yuan^3^, Jianjun Zhang^1#^, Kang He^1#^, Qiang Xia^1,4,5#^

^1^ Department of Liver Surgery, Renji Hospital, School of Medicine, Shanghai Jiao Tong University, Shanghai, China

^2^ Department of Orthopedics, Ruijin Hospital, Shanghai Jiaotong University School of Medicine, Shanghai, China

^3^ Department of Gastroenterology, Hepatology and Endocrinology, Hannover Medical School, Hannover, Germany

^4^ Shanghai Engineering Research Center of Transplantation and Immunology, Shanghai, China

^5^ Shanghai Institute of Transplantation, Shanghai, China

*** Correspondence:**

Jianjun Zhang
[zhangjianjun0221@126.com](mailto:zhangjianjun@renji.com)

Kang He

[hekang929@163.com](mailto:hekang929@163.com)

Qiang Xia

[xiaqiang@shsmu.edu.cn](mailto:xiaqiang@shsmu.edu.cn)

Keywords: Virus-like Mesoporous Silica, Bionic Inspired Nanosystem, Precise Delivery of mRNA, Liver Target, Oral in Situ Injection

# Supplementary Figures


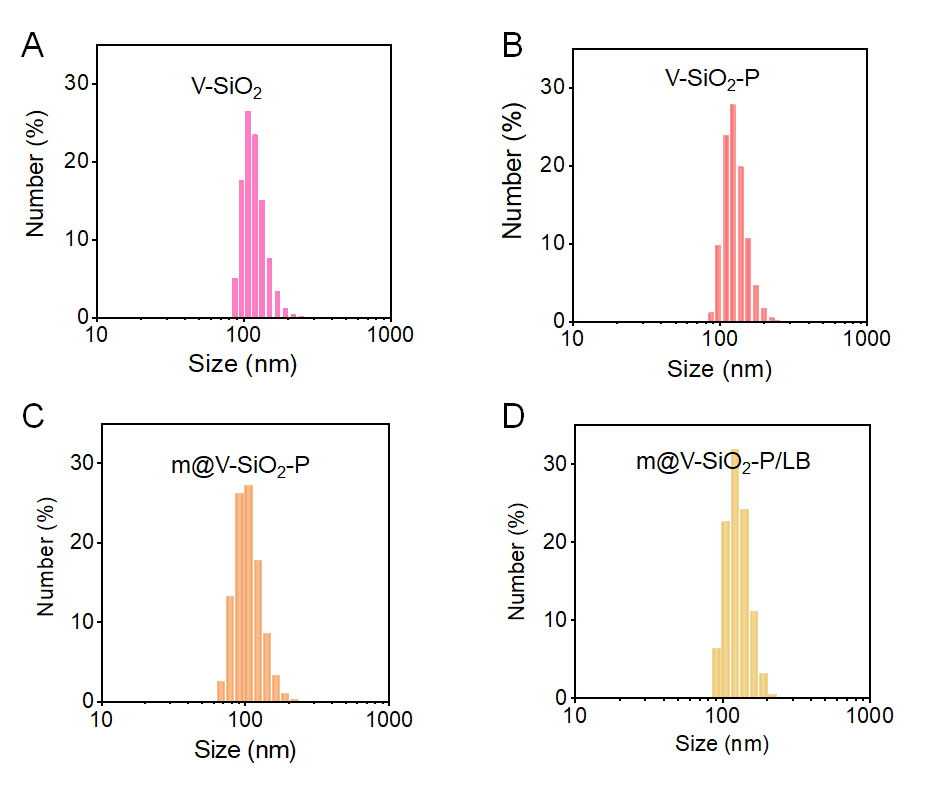


**Supplementary Figure 1.** Size distribution of V-SiO_2_ (A), V-SiO_2_-P (B), m@V-SiO_2_-P (C) and m@V-SiO_2_-P/LB nanoparticles (D) by DLS analysis.


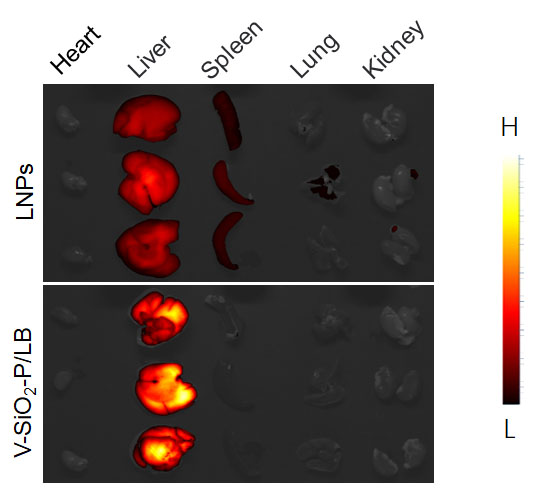


**Supplementary Figure 2.** *Ex vivo* fluorescence signals of major organs at 48 h after injection.
